# The efficacy and safety of adjunctive corticosteroids in the treatment of tuberculous pleurisy: a systematic review and meta-analysis

**DOI:** 10.18632/oncotarget.18160

**Published:** 2017-05-24

**Authors:** Shuanshuan Xie, Lin Lu, Ming Li, Mengting Xiong, Shunping Zhou, Guoliang Zhang, Aimei Peng, Changhui Wang

**Affiliations:** ^1^ Department of Respiratory Medicine, Shanghai Tenth People’s Hospital, Tongji University, Shanghai, China; ^2^ Department of Nephrology, North Huashan Hospital, Fudan University, Shanghai, China; ^3^ Department of Cardiology Medicine, Pudong Hospital, Fudan University, Shanghai, China; ^4^ Department of Cardiology Medicine, Yangpu Hospital, Tongji University School of Medicine, Shanghai, China

**Keywords:** tuberculous pleurisy, corticosteroid, pleural fluid, pleural thickening, pleural adhesion

## Abstract

**Purpose:**

To evaluate the efficacy and safety of adjunctive corticosteroids in the treatment of patients with tuberculous pleurisy.

**Methods:**

The PubMed, Cochrane, Medline, Embase, Web of Science and Chinese National Knowledge Infrastructure were searched. Clinical trials of corticosteroids compared with control were eligible for inclusion.

**Results:**

Ten studies (6 randomized controlled trials [RCTs] and 4 non-RCTs) with 957 participants met the inclusion criteria. Compared to the controls (placebos or non-steroids), adjunctive corticosteroid use reduced the risk of residual pleural fluid after 4 weeks and the number of days to symptom improvement; however, there was no convincing evidence to support the positive effects of corticosteroids over the long term (8 weeks) on residual pleural fluid, pleural thickening, or pleural adhesions, and there was no statistical difference between the corticosteroid group and control group with respect to 7-days relief of the clinical symptoms or death from any cause. In addition, more adverse events were observed in patients who received corticosteroids than in those in the control group.

**Conclusions:**

Our results suggest that adjunctive corticosteroid use did not improve long-term efficacy and might induce more adverse events, although the risk of residual pleural fluid at 4 weeks and the number of days to symptom improvement were reduced.

## INTRODUCTION

Tuberculosis (TB) is a serious infectious disease that results in 2.0 million deaths per year [1]. Although pulmonary TB is the most common form of TB infections, extrapulmonary tissues are often infected as well [2]. For example, tuberculous pleurisy is common in extrapulmonary TB [3] and accounts for ∼4.0% of all TB cases in western countries and ∼20% of all TB cases in South Africa [4-6]. Despite 6.0–9.0 months of anti-TB drug treatment, tuberculous pleurisy might still result in pleural fibrosis, calcification, and thickening. To prevent these complications, corticosteroids are frequently used in addition to conventional anti-TB drugs.

The efficacy and safety of corticosteroids on tuberculous pleurisy were conflicting. Two non-randomized controlled trials (RCTs) [7, 8] showed that corticosteroids could promote pleural fluid absorption and reduce pleural thickening; however, Galarza et al [9] demonstrated that no significant difference in the lung capacity, pleural sequelae, and pleural fluid reabsorption rate between corticosteroid and placebo group. In addition, a non-RCT conducted by Mansour et al [10] also showed that the difference was not statistically significant after 6 months; therefore, the effect of adjunctive corticosteroids on tuberculous pleural effusion remains uncertain and controversial.

In this study, the potential benefits and detriments of corticosteroids in treating tuberculous pleural effusion were assessed by reviewing and analyzing RCTs and non-RCTs.

## RESULTS

### Search of the published literature

**Figure 1:**
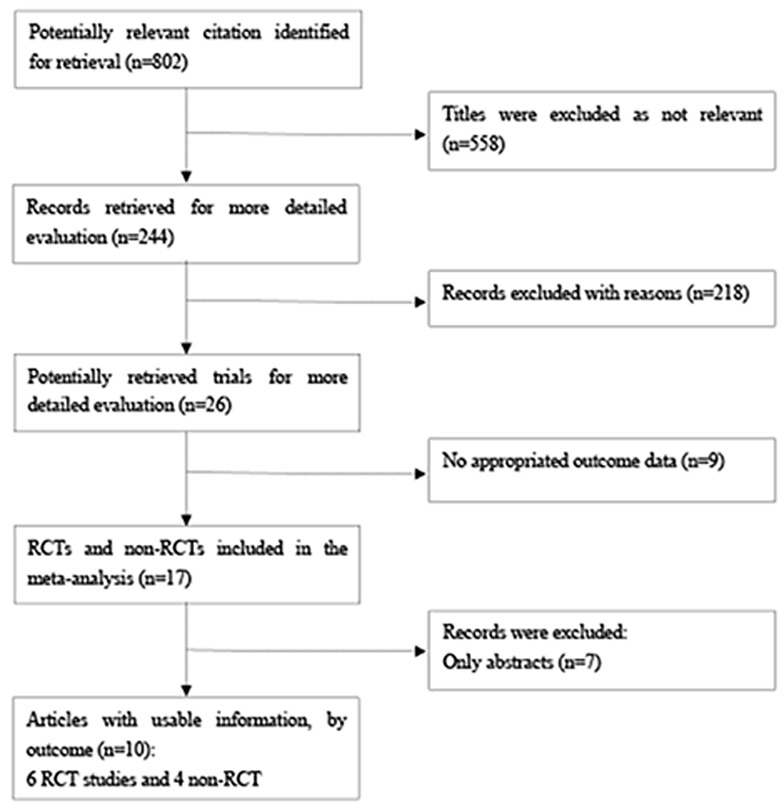
Procedures used for trial selection Abbreviations: RCT, randomized controlled trial; Non-RCT, non-randomized controlled trial.

The systematic literature search identified 802 studies on corticosteroids, of which 17 trials comprised patients with tuberculous pleurisy. After excluding 7 studies, 10 trials were included in this analysis that involved 957 tuberculous pleurisy patients [8-10, 18-24]. Figure 1 and Supplementary File 1 show the reasons for the exclusion of various studies.

Bias risk of each item for the included RCTs is shown in Supplementary Figure S1 and Table S1. Only Lee et al [20]. reported “high risk” in Free of the selected reporting items shown in Supplementary Figure 2. The trial results were analyzed for sensitivity by removing the items of high-risk bias. The quality scores of non-RCTs are summarized in Supplementary Table 2. Most of the observational studies suggested reasonably good-quality RCTs with a score of 5 or higher on the Jadad scale.

### Studies included in the assessment

Ten studies on corticosteroid use (six randomized studies and four cohort studies) between 1958 and 2006 were included in the study (Table 1) and comprised 957 patients [8-10, 18-24]. Of these 10 studies, two were conducted in South Korea [18, 21], and two in India [8, 24]; the others were conducted in Uganda [19], South Africa [22], Taiwan [20], England [23], and Iraq [10]. Nine trials comprised both men and women; 62% (men, from 47 to 97%). The follow-up period ranged from 6.0 months [22] to 46 months [9]. Nine studies included prednisone or placebos as an adjunct to the anti-TB regimen, including INH (isoniazid), RM (rifampin), PZA (pyrazinamide), EMB (ethambutol), SM (streptomycin) (except in Mansour, which did not state them in detail) [8, 9, 18-24]. Corticosteroids were given orally in various doses (0.75–1.0 mg/kg/day) [8-10, 19, 20, 22, 23]. The patients in the Bang and Lee [18, 21] studies were injected with 1.0 mg/kg/d and 30 mg/d corticosteroids, and the patients in Mansour et al [24] were treated with 125–250 mg intrapleural hydrocortisone.

**Table 1:** Characteristics of studies included in the meta-analysis

Study	Year	Study period	Location	No of cases	Age (mean)	Gender Male (%)	Anti-TB regimen	Corticosteroid
Lee [20]	1988	1983-1987	Taiwan	40	29	60%	INH 300 mg/d	Prednisolone 0.75 mg/kg/d po
							RM 450 mg/d	
							EMB 20 mg/kg/d	
Galarza [9]	1995	1985-1992	Spain	117	27	51%	INH 300 mg (5mg/kg/d)	Prednisolone 1 mg/kg/d po
							RM 600 mg/d (10 mg/kg/d)	
Wyser [22]	1996	1994-1995	South Africa	70	33	61%	EMB 10 mg/kg/d	Prednisolone 0.75 mg/kg/d po
							INH 8 mg/kg/d	
							PZA 25 mg/kg/d	
							Pyridoxine 25 mg/kg/d	
Bang [18]	1997	1991-1994	Korea	83	34	59%	INH 400 mg/d	Prednisolone 1 mg/kg/d injection
							RM 600 mg/d	
							PZA 1500 mg/d	
							EMB 800 mg/d	
Lee [21]	1999	1990-1997	Korea	82	32	64%	INH (NS)	Prednisolone 30 mg/d injection
							RM	
							PZA	
							EMB	
							Streptomycin	
Elliott [19]	2004	1988-2002	Uganda	194	34	58%	EMB 20 mg/kg/d	Prednisolone 50, 40, 25, 15 mg/d (respectively 14 days) po
							INH 5 mg/kg/d	
							RM 10 mg/kg./d	
							PZA 18-26 mg/kg/d	
Aspin [23]	1958	1955-1957	England	30	NA	NA	INH 300 mg/d	ACTH 40 units/d or prednisone 20 mg/d po
							SM 1g/d	
Menon [8]	1964	1959-1962	India	49	25 and >25	47%	INH 200 mg/d	Intrapleural
							SM 1g/d	Hydrocortisone 25 mg/d or prednisolone 15 mg/d/ po
Mathur [24]	1965	1958-1962	India	102	4-75	62%	INH 300 mg/d	Intrapleural
							SM 1g/d	Hydrocortisone 125-250 mg
Mansour [10]	2006	2003-2004	Iraq	190	17-45	97%	Anti-TB (NS)	Prednisolone 30mg/d po

Abbreviation: INH, isoniazid; SM, streptomycin; PAS, p-amino-salicylic acid; RM, rifampin; EMB, ethambutol; PZA, pyrazinamide; NS, not stated in detail

The outcomes comprised pleural effusion reabsorption (six studies reported the outcome at 4.0 weeks [8, 9, 18-20, 24] and six studies reported the outcome at 8.0 weeks, [8, 18-21]), the presence of pleural thickening (seven trials), [8-10, 20-22, 24] and adhesions (two trials) [18, 20] number of days to achieve improved symptoms and signs (three trials), [10, 18, 20], 7-d relief of clinical symptoms (two trials), [20, 24] death from any cause (one trial), [19] and adverse effects (seven trials).[9, 18-22, 24]

### Primary outcomes

**Figure 2:**
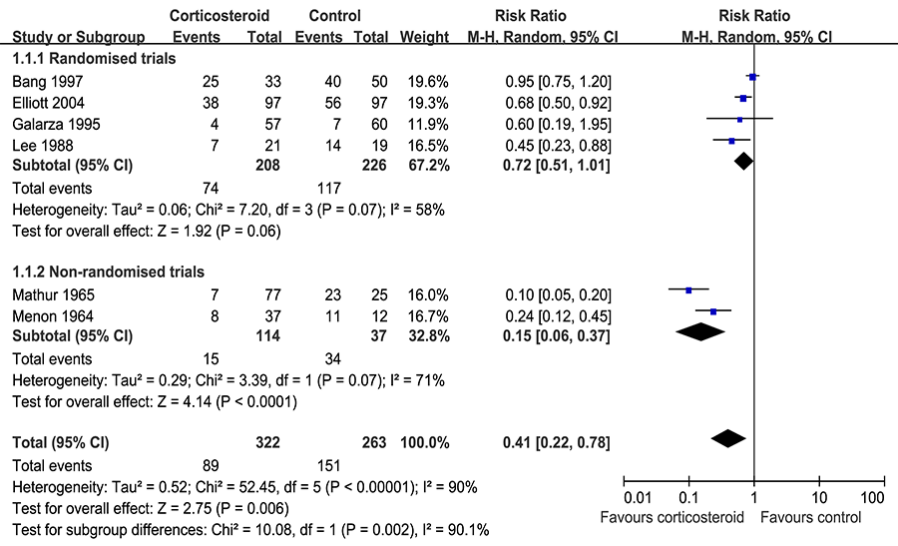
Results of the meta-analysis on studies evaluating adjunctive corticosteroid use on reabsorption of pleural effusion at 4.0 weeks: response rate = 0.41 (95% confidence interval: 0.22–0.78)

**Figure 3:**
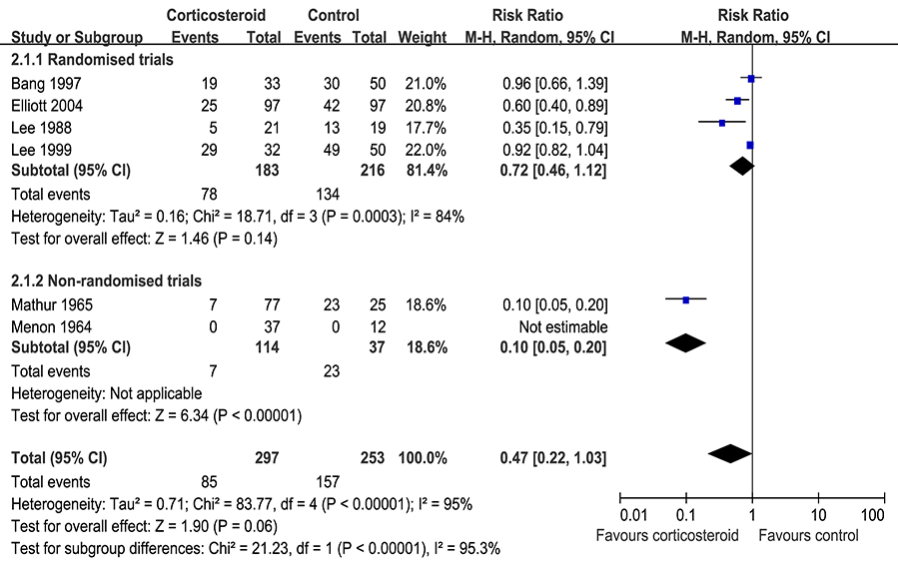
Adjunctive corticosteroid uses on reabsorption of pleural effusion at 8.0 weeks: response rate = 0.47 (95% confidence interval: 0.22–1.03)

Four RCTs and two non-RCTs comprising 585 cases reported pleural effusion reabsorption at 4.0 weeks [8, 9, 18-20, 24]. Compared to placebos or non-corticosteroids, adjunctive corticosteroid use reduced the residual fluid at 4.0 weeks (RR = 0.41, 95% CI: 0.22–0.78, *p* = 0.006) (Figure 2). In addition, six trials reported pleural effusion reabsorption at 8.0 weeks in 550 patients. Our study showed that corticosteroids did not appear to provide statistically significant benefits for reducing residual pleural effusion after 8.0 weeks in the treatment of tuberculous pleurisy (RR = 0.47, 95% CI: 0.22–1.03, *P* = 0.06) (Figure 3).

Seven trials reported pleural thickening in 650 patients. There was no statistically significant difference between the corticosteroid and non-corticosteroid group (RR = 0.97, 95% CI: 0.77–1.23, *p* = 0.83) (Supplementary Figure 3). Two RCTs comprising 53 cases also reported no difference in pleural adhesions (RR = 0.75, 95% CI: 0.51–1.11, *p* = 0.15) (Supplementary Figure 4).

### Secondary outcomes

**Figure 4:**
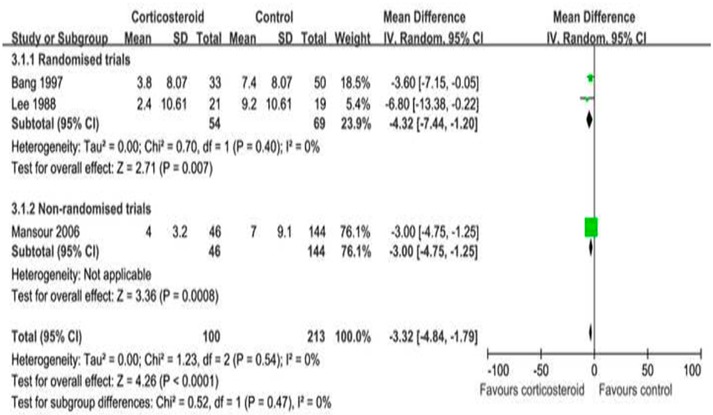
Adjunctive corticosteroid uses on the number of days of clinical symptoms: weighted mean difference = -3.32 (95% confidence interval: -4.84–-1.79)

Three included trials [10, 18, 20] reported that corticosteroids could decrease the number of days of clinical symptoms (WMD =-3.32, 95% CI: -4.84–-1.79, *p* < 0.0001) (Figure 4). In addition, two trials [20, 24] that reported 7-d clinical symptoms concluded that there was no difference in results between the corticosteroid and non-corticosteroid groups (RR = 0.87, 95% CI: 0.00–162.23, *p =* 0.96) (Supplementary Figure 5).

### Adverse events

**Figure 5:**
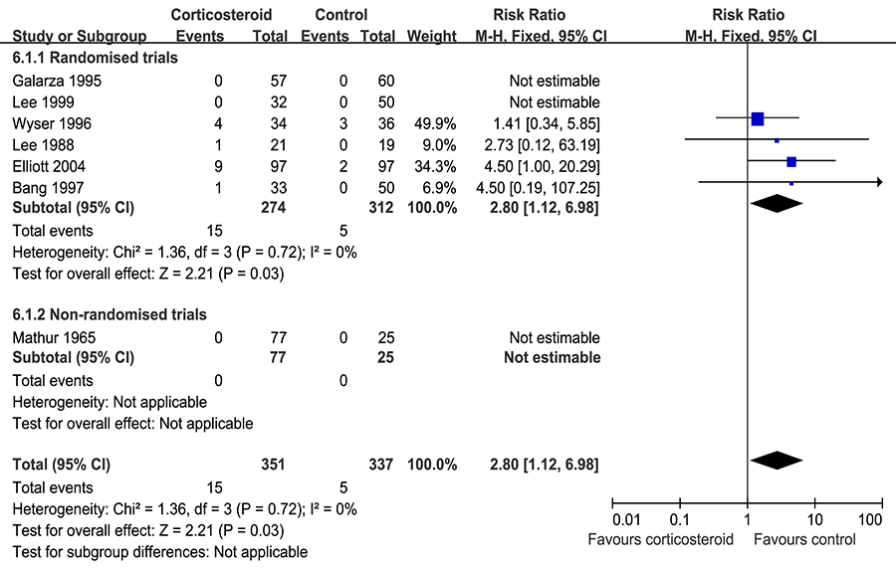
Adverse effects of adjunctive corticosteroid: response rate = 2.80 (95% confidence interval: 1.12–6.98)

The risk of adverse events (AEs) between the corticosteroid and control groups in patients with tuberculous pleurisy was assessed. Six RCTs and one non-RCT comprising 688 cases examined the statistical difference between the groups on the risk of AE (RR = 2.80, 95% CI: 1.12–6.98, *p* = 0.03) (Figure 5). The risk of adverse events (8%) were existed on corticosteroid group as compared with patients who did not receive corticosteroid.

Limited data are available regarding the effects of corticosteroids on mortality in tuberculous pleurisy. Only one trial assessed the risk of death and found that the mortality rate was not significantly decreased in patients using corticosteroids (RR = 0.92, 95% CI 0.65–1.32).

In addition, only one trial [19] reported the effect of corticosteroids on HIV-associated infections (gastroenteritis, cryptococcal meningitis, esophageal candidiasis, herpes simplex, herpes zoster, and oral thrush) and found that the result was not significantly different in patients using corticosteroids (RR = 13.00, 95% CI 0.74–227.63).

### Sensitivity analysis and publication bias

The above results did not change by using the random-effects model and fixed-effects model. The funnel plot of all items are shown in the Supplementary Information (Supplementary Figures 6–12). The Egger’s and Begg’s tests showed no publication bias (*P* = 0.12 and *P* = 0.21, respectively).

## DISCUSSION

This systematic review suggests that adjunctive corticosteroid use did not appear to exhibit improved long-term efficacy and might induce more detrimental effects, although the risk of residual pleural fluid at 4.0 weeks and the number of days to symptom improvement were reduced.

The conclusions drawn from different studies about using corticosteroids in addition to anti-TB drugs in patients with tuberculous pleurisy have been inconsistent. The first related RCT conducted by Lee et al [20], 40 patients with tuberculous pleurisy showed that corticosteroid administration could relieve the clinical symptoms and decrease pleural effusion (*p* < 0.05) compared to those in the placebo group, but their study failed to confirm whether corticosteroid use reduced pleural thickening; however, a non-RCT conducted by Mansour et al [10], 190 patients with tuberculous pleural effusion showed that the results were no longer statistically significant after 6.0 months and they concluded that corticosteroid therapy was unnecessary in the management of tuberculous pleural effusion. A previous systematic review comprising six RCTs and 633 patients conducted by Engel et al [25]. suggested that corticosteroid use was concerned with absorption of residual pleural fluid after 4.0 weeks (RR = 0.76, 95% CI: 0.62–0.94, *p* = 0.0095) and reduced pleural thickening (RR = 0.69, 95% CI: 0.51–0.94, *p* = 0.019). An overview of the above results indicates that there is no strong evidence to assess whether steroids are effective in the treatment of tuberculous pleurisy. In our meta-analysis, we found no proof to favorably support the effect of corticosteroids on residual pleural fluid after 8.0 weeks (RR = 0.72, 95% CI: 0.46–1.12, *p* = 0.14) and pleural adhesions in patients with tuberculous pleurisy (RR = 0.75, 95% CI: 0.51–1.11, *p* = 0.15), and the effect of corticosteroids on pleural thickening was also insignificant (RR = 0.97, 95% CI: 0.77–1.23, *p* = 0.83). Moreover, corticosteroids had a detrimental adverse effect on patients with tuberculous pleurisy (RR = 2.80, 95% CI: 1.12–6.98, *p* = 0.03). This difference between the two studies could be explained by the inclusion of non-RCTs in our meta-analysis.

The detail Resolution of symptoms: dyspnoea, cough, night sweats, tiredness, appetite, pleuritic chest pain, and general well-being were each graded from 0 to 100 using a visual analogue scale and combined index with a maximum score of 700 was calculated. Because of the lack of consistent results, more trials are urgently needed to evaluate the effect of corticosteroids on symptoms. We reanalyzed the number of days of clinical symptoms and found that the time to disappearance of symptoms was significantly shorter in participants who received corticosteroids (WMD =-3.32, 95% CI: -4.84–-1.79, *p* < 0.0001). In addition, Lee [20] demonstrated that fever, dyspnea, and chest pain were more likely to be resolved by 7 d after treatment in patients who received corticosteroids compared to those who received the placebo.

Potential adverse effects of prednisone were well recognized and occurred in the corticosteroids group (Supplementary Table 3). For example, in the study of Elliott et al [19] treatment was discontinued in 9 of the 97 patients in both groups because of the onset of hyperglycemia, hypertension, infections, and other adverse effects. Wyser et al [22]. reported epigastric pain in 4 of the 34 cases in the corticosteroid group and 3 of the 36 cases in the control group. Lee et al [20, 21]. reported epigastric pain in one case in the corticosteroid group but none in the non-corticosteroid group, and Bang et al [18]. reported epigastric pain in one case in the corticosteroid group but none in the non-corticosteroid group. In general, more patients experienced AEs in the corticosteroid group.

This meta-analysis is the latest to examine the effect of corticosteroids in the treatment of tuberculous pleurisy. First, it considered the difference in the design of corticosteroids and traditional therapy in tuberculous pleurisy. Second, it combined data from both RCTs and non-RCTs, thus significantly increasing statistical reliability. Finally, the results remained unchanged on sensitivity analysis. Limitations of our review were that this meta-analysis included only published studies in indexed journals and did not consider unpublished studies; however, no evidence of substantial publication bias was found.

## CONCLUSIONS

Our results suggest that adjunctive corticosteroid use does not improve the long-term efficacy of tuberculous pleurisy and might lead to detrimental adverse effects, although the ratio of residual pleural fluid (4.0 weeks) to the number of days for symptom improvement was reduced. Thus, corticosteroid treatment might not be necessary in the management of tuberculous pleurisy, and clinicians should make a decision for use by justifying the risk-to-benefit ratio of adjunctive corticosteroid treatment in patients with tuberculous pleurisy.

## MATERIALS AND METHODS

### Search strategy

The data were extracted from quality articles in PubMed, Cochrane, Medline, Embase, Web of Science, Chinese National Knowledge Infrastructure, and reference lists, and by manual searches in July 2015, using the following terms: ‘tuberculous pleurisy’, placebo, ‘‘corticosteroids, “steroids, ‘TB, ‘residual fluid, pleural thickening, clinical symptoms, ‘adverse events (AE), pleural adhesions.

### Data extraction and quality assessment

Trials were excluded if they did not meet with the following inclusion criteria. The following inclusion criteria were used for determining which trials to use in the study: 1) trials that compared corticosteroids with a control (placebo or no steroids); 2) trials that enrolled tuberculous pleurisy cases; and 3) trials that reported results on residual fluid, pleural thickening, pleural adhesions, clinical symptoms, adverse events, and death. Two independent investigators (AP and SX) assessed each inclusion trial and extracted the data, such as trial characteristics and outcome measures (e.g., pleural thickening, pleural adhesions, adverse events, *p* values, response rate [RR] for residual fluid, 95% confidence interval [CI], and weighted mean difference [WMD] for number of days of clinical symptoms). The quality of RCTs was assessed using the Jadad scale, and the non-RCT studies were estimated using the 9-star Newcastle–Ottawa Scale [11, 12]. Details of exactly data were extracted from each article in Supplementary Table 2.

A list of excluded articles and reasons of exclusion were showed in Supplementary File 1 and Figure 1.

### Statistical analyses

The outcomes were analyzed by Review Manager 5.0. *x*^*2*^ and *I*^*2*^ tests were used to assess whether the outcome of the trials had heterogeneity [13]. *p* < 0.1 or *I*^*2*^ > 50% was considered heterogeneous. [14] If heterogeneity was detected, the random-effects model was performed to analyze the outcomes [15]. In addition, funnel plots and the Egger’s and Begg’s tests were performed to evaluate publication bias [16]. The trial results were analyzed for sensitivity by removing the items of “high-risk bias” [17].

## SUPPLEMENTARY MATERIALS FIGURES AND TABLES







## Abbreviations

RCT: Randomized controlled trial
TB: Tuberculosis
AE: Adverse event
RR: Response rate
CI: Confidence interval
WMD: weighted mean difference
INH: soniazid
RM: rifampin
PZA: pyrazinamide
EMB: ethambutol
SM: streptomycin

## References

[1] World Health Organization

[2] Harries AD (1990). Tuberculosis and human immunodeficiency virus infection in developing countries. Lancet.

[3] Light RW (2010). Update on tuberculous pleural effusion. Respirology.

[4] Baumann MH, Nolan R, Petrini M, Lee YC, Light RW, Schneider E (2007). Pleural tuberculosis in the United States: incidence and drug resistance. Chest.

[5] Seiscento M, Vargas FS, Rujula MJ, Bombarda S, Uip DE, Galesi VM (2009). Epidemiological aspects of pleural tuberculosis in the state of Sao Paulo, Brazil (1998-2005). J Bras Pneumol.

[6] Saks AM, Posner R (1992). Tuberculosis in HIV positive patients in South Africa: a comparative radio logical study with HIV negative patients. Clin Radiol.

[7] Singh D, Yesikar SS (1965). Role of intrapleural corticosteroids in tuberculous pleural effusion: a clinicotherapeutic trial of 50 cases. J Indian Med Assoc.

[8] Menon NK (1964). Steroid therapy in tuberculous effusion. Tubercle.

[9] Galarza I, Canete C, Granados A, Estopa R, Manresa F (1995). Randomised trial of corticosteroids in the treatment of tuberculous pleurisy. Thorax.

[10] Mansour AA, Al-Rbeay TB (2006). Adjunct therapy with corticosteroids or paracentesis for treatment of tuberculous pleural effusion. East Mediterr Health J.

[11] Higgins JP, Green S www.cochrane-handbook.org.

[12] Stang A (2010). Critical evaluation of the Newcastle-Ottawa scale for the assessment of the quality of nonrandomized studies in meta-analyses. Eur J Epidemiol.

[13] Higgins JP, Thompson SG (2002). Quantifying heterogeneity in a meta-analysis. Stat Med.

[14] Parmar MK, Torri V, Stewart L (1988). Extracting summary statistics to perform meta-analyses of the published literature for survival endpoints. Stat Med.

[15] Schulz KF, Chalmers I, Hayes RJ, Altman DG (1995). Empirical evidence of bias. Dimensions of methodological quality associated with estimates of treatment effects in controlled trials. JAMA.

[16] DerSimonian R, Laird N (1986). Meta-analysis in clinical trials. Control Clin Trials.

[17] Egger M, Davey Smith G, Schneider M, Minder C (1997). Bias in meta-analysis detected by a simple, graphical test. BMJ.

[18] Bang JS, Kim MS, Kwak SM, Cho CH (1997). Evaluation of steroid therapy in tuberculous pleurisy: A prospective, randomized study. Tuberculosis and Respiratory Disease.

[19] Elliott AM, Luzze H, Quigley MA, Elliott AM, Luzze H, Quigley MA (2004). A randomized, double-blind, placebo-controlled trial of the use of prednisolone as an adjunct to treatment in HIV-1-associated pleural tuberculosis. J Infect Dis.

[20] Lee CH, Wang WJ, Lan RS, Tsai YH, Chiang YC (1988). Corticosteroids in the treatment of tuberculous pleurisy. A double-blind, placebo-controlled, randomized study. Chest.

[21] Lee BH, Jee HS, Choi JC, Park YB, Ahn CH, Kim JY, Park IW, Choi BW, Hue SH (1999). Therapeutic effect of prednisolone in tuberculous pleurisy: A prospective study for the prevention of the pleural adhesion. Tuberculosis and Respiratory. Disease.

[22] Wyser C, Walzl G, Smedema JP Swart F, van Schalkwyk EM, van de Wal BW (1996). Corticosteroids in the treatment of tuberculous pleurisy. A double-blind, placebo-controlled, randomized study. Br J Tuberc Dis Chest.

[23] Aspin J, O’Hara H (1958). Steroid-treated tuberculous pleural effusions. Br J Tuberc Dis Chest.

[24] Mathur KS, Mathur JS, Sapru RP (1965). Treatment of tuberculosis pleural effusion with local instillation of hydrocortisone. Dis Chest.

[25] Engel ME, Matchaba PT, Volmink J (2007). Corticosteroids for tuberculous pleurisy. Cochrane. Cochrane Database Syst Rev.

