# Supplementary material for: The efficacy and safety of adjunctive corticosteroids in the treatment of tuberculous pleurisy: a systematic review and meta-analysis
Source: Oncotarget. 2017 May 24;8(47):83315–22. doi: 10.18632/oncotarget.18160 (PMC5669971; doi:10.18632/oncotarget.18160)
Supplement: Supplementary file 1 [file oncotarget-08-83315-s001.pdf]

The efficacy and safety of adjunctive corticosteroids in the treatment of tuberculous pleurisy: a systematic review and meta-analysis

SUPPLEMENTARY MATERIALS

Additional Files in Supplementary Files
Additional File 1: Search strategies: A list of included and excluded articles and reasons of exclusion
Additional File 2: PRISMA checklist

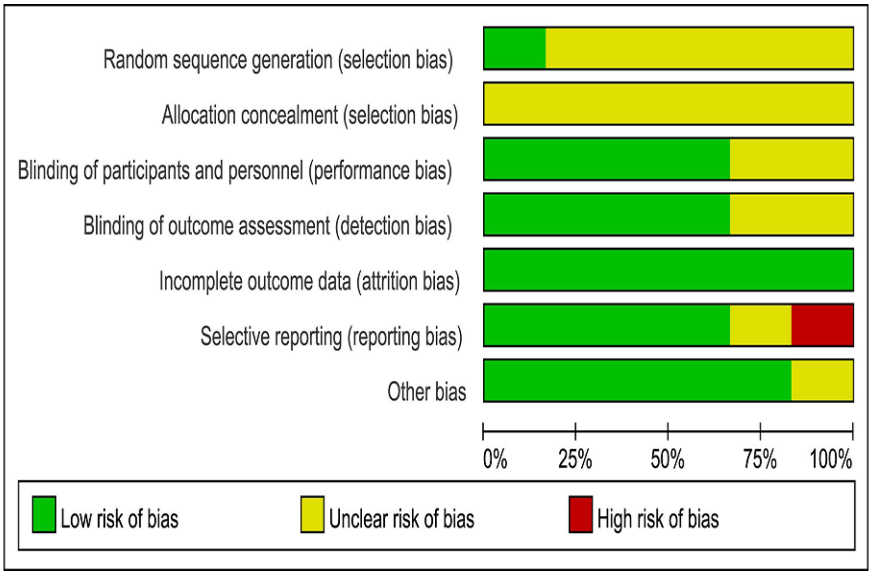

Supplementary Figure 1: Summary of the bias risk in included randomized controlled trials.

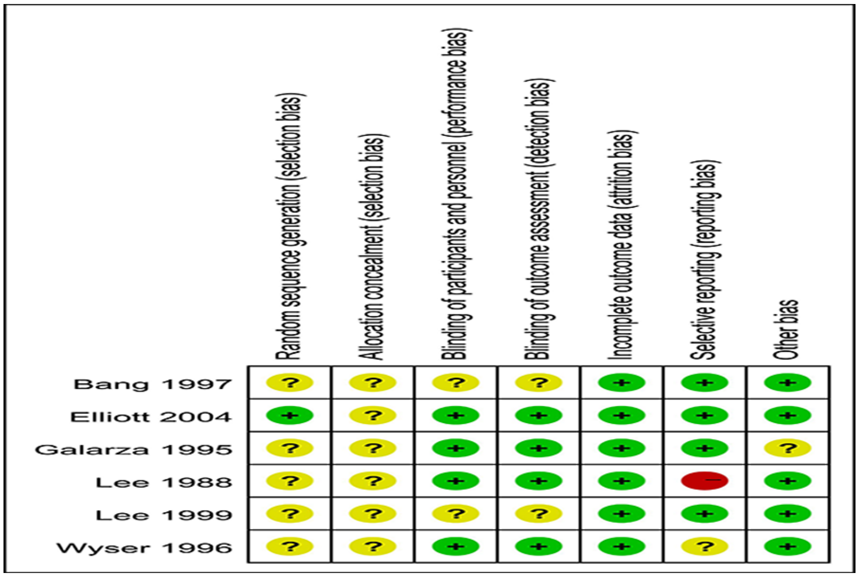

Supplementary Figure 2: Results of the bias risk with each item in included randomized controlled trials.

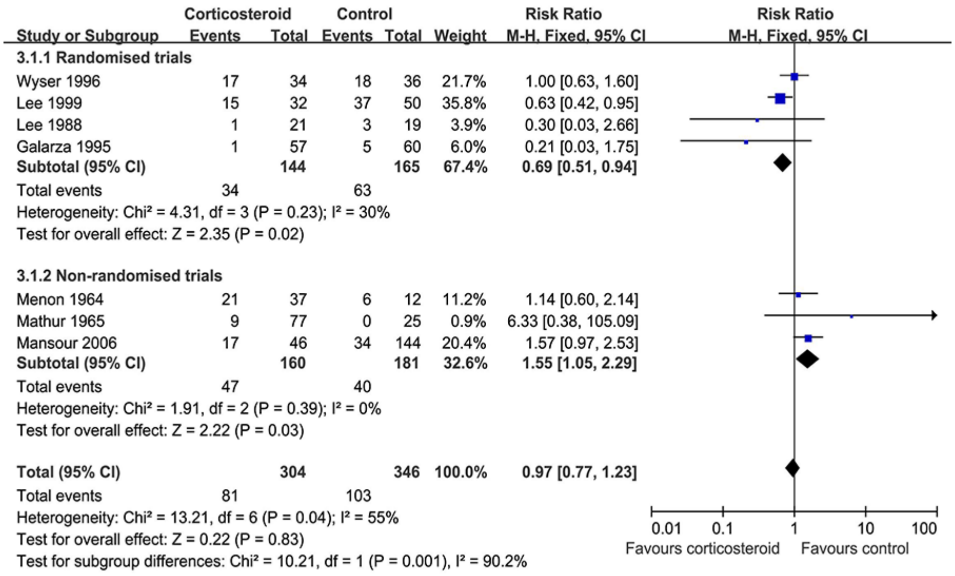

Supplementary Figure 3: Adjunctive corticosteroid uses on pleural thickening: response rate = 0.97 (95% confidence interval: 0.77–1.23).

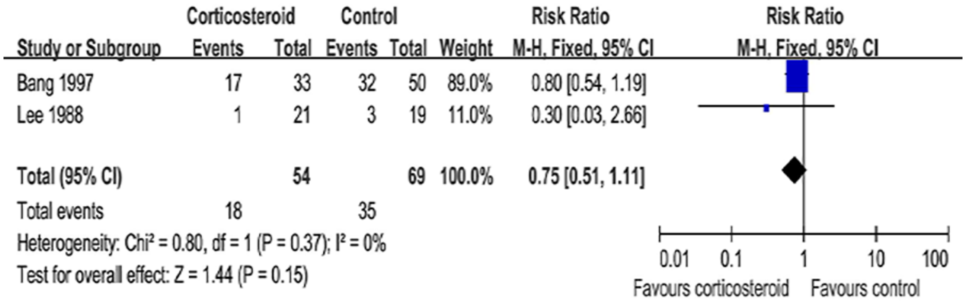

Supplementary Figure 4: Adjunctive corticosteroid uses on pleural adhesions: response rate = 0.75 (95% confidence interval: 0.51–1.11).

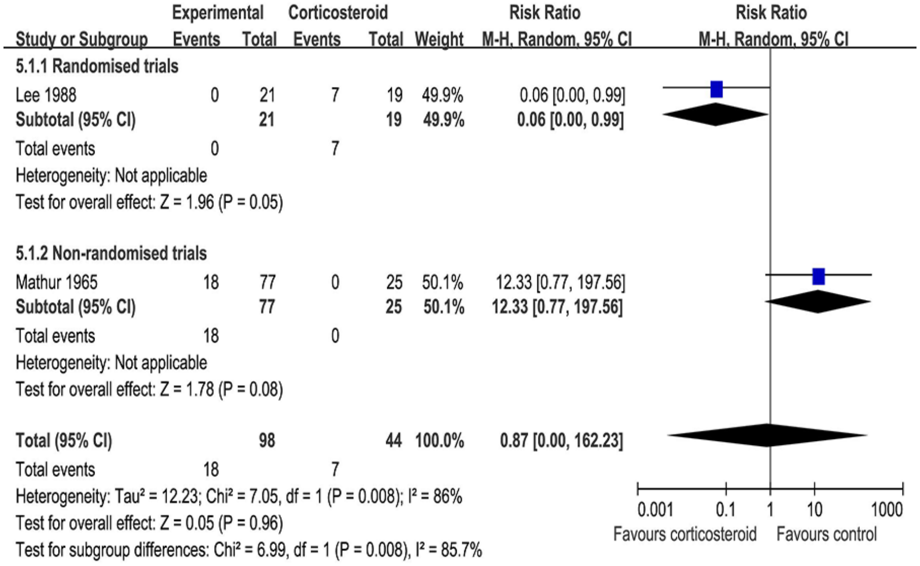

Supplementary Figure 5: Adjunctive corticosteroid uses on clinical symptoms after 7.0 d: response rate = 0.87 (95% confidence interval: 0.00–162.23).

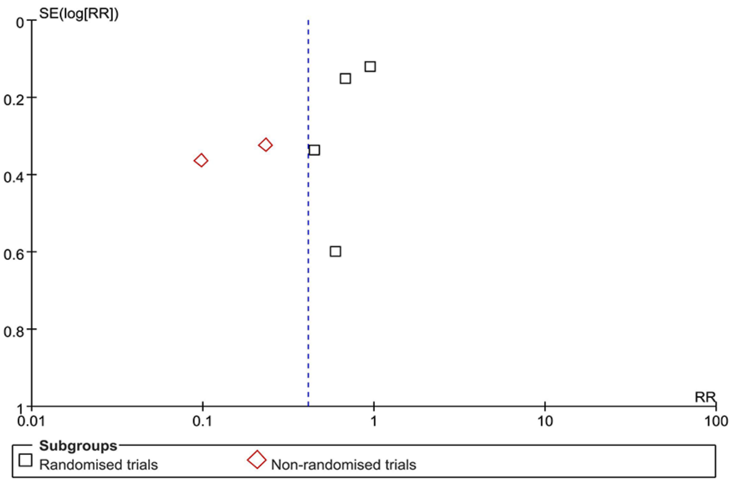

Supplementary Figure 6: Adjunctive corticosteroid uses on reabsorption of pleural effusion at 4.0 weeks. RR: response rate.

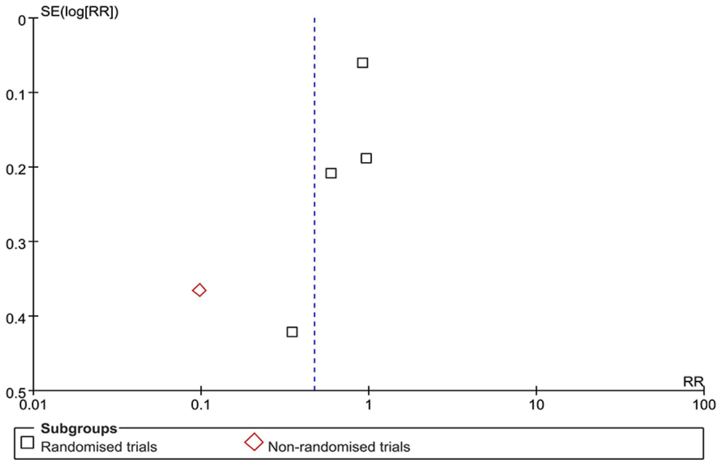

Supplementary Figure 7: Adjunctive corticosteroid uses on reabsorption of pleural effusion at 8.0 weeks. RR: response rate.

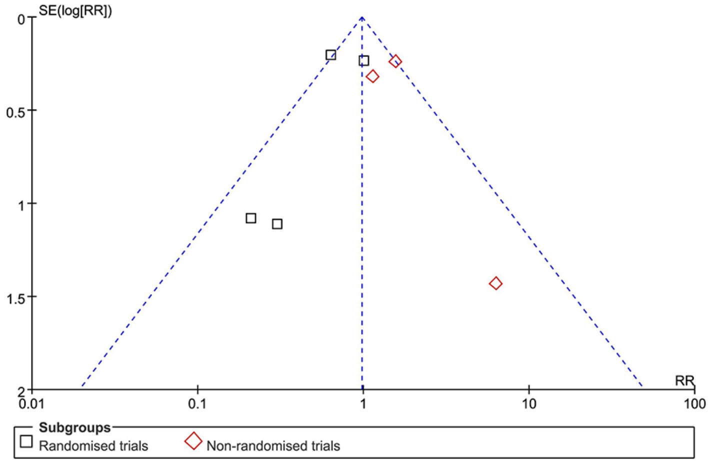

Supplementary Figure 8: Adjunctive corticosteroid uses on pleural thickening. RR: response rate.

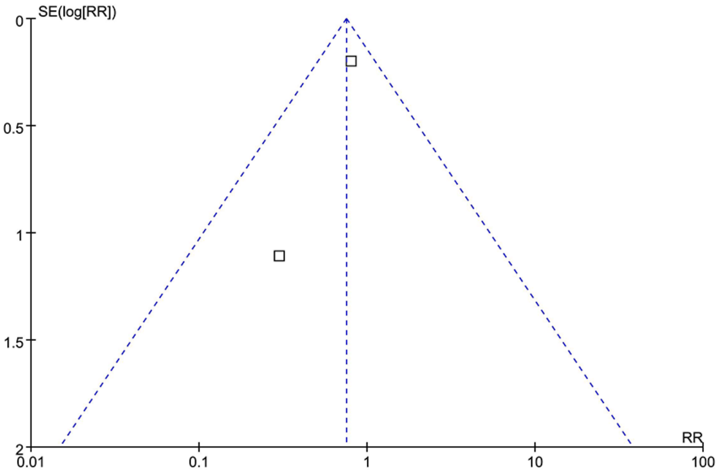

Supplementary Figure 9: Adjunctive corticosteroid uses on pleural adhesions. RR: response rate.

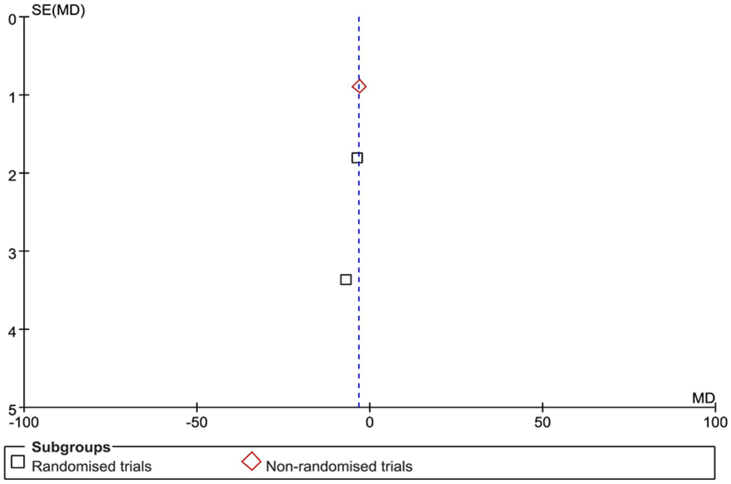

Supplementary Figure 10: Adjunctive corticosteroid uses on the number of days of clinical symptoms. WMD: weighted mean difference.

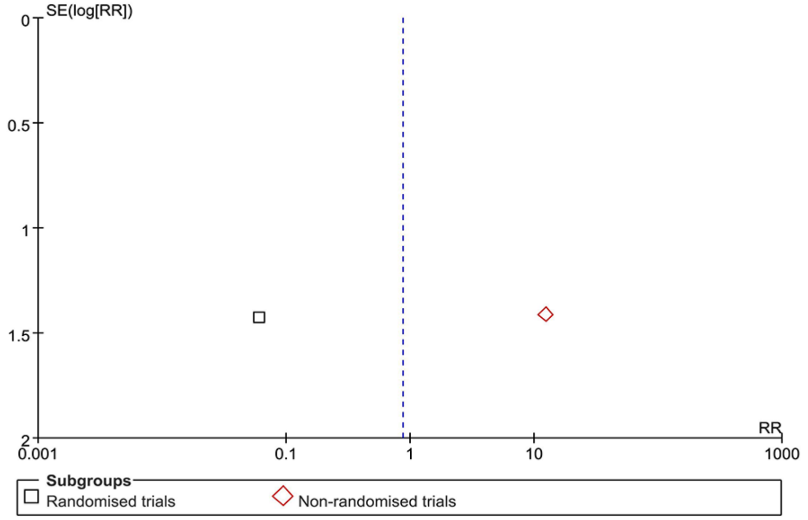

Supplementary Figure 11: Adjunctive corticosteroid uses on clinical symptoms after 7.0 d. RR: response rate.

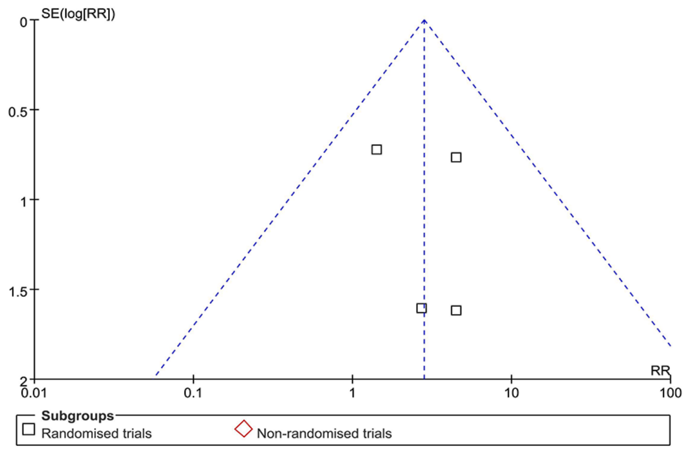

Supplementary Figure 12: Adjunctive corticosteroid uses on adverse events. RR: response rate.

Supplementary Table 1: Corticosteroids versus control (placebo or no steroids)

Outcome or subgroup title	No. of studies	No. of participants	Statistical method	Effect size
Residual fluid at 4 weeks	6	585	Risk Ratio (M-H, Fixed, 95% CI)	0.54 [0.45, 0.64] asymmetric
Residual fluid at 8 weeks	6	550	Risk Ratio (M-H, Fixed, 95% CI)	0.60 [0.51, 0.71] asymmetric
Presence of pleural thickening	7	650	Risk Ratio (M-H, Fixed, 95% CI)	0.97 [0.77, 1.23] asymmetric
Presence of pleural adhesions	2	123	Risk Ratio (M-H, Fixed, 95% CI)	0.75 [0.51, 1.11] asymmetric
Days to improvement in symptoms	3	313	Mean Difference (IV, Random, 95% CI)	-3.32 [-4.84, -1.79] symmetric
Clinical symptoms after 7 days	2	142	Risk Ratio (M-H, Random, 95% CI)	0.87 [0.00, 162.23] symmetric
Adverse events	7	688	Risk Ratio (M-H, Fixed, 95% CI)	2.80 [1.12, 6.98] symmetric
Death from any cause	1	194	Risk Ratio (M-H, Fixed, 95% CI)	0.92 [0.65, 1.32]
HIV-associated events	1	97	Risk Ratio (M-H, Fixed, 95% CI)	13.00 [0.74 , 227.63]

Supplementary Table 2: Methodological quality of included case–control studies based on the Newcastle–Ottawa Scale.									
Case-control studies	Selection				Comparability	Exposure			**Total score**
	Adequate definition of cases	Representative of cases	Selection of controls	Definition of controls	Control for important factor or additional factor	Ascertainment of exposure	Same method of ascertainment for cases and controls	Non-response rate	
Aspin [23]		*	*	*		*	*		**5**
Menon [8]	*	*	*	*		*	*		**6**
Mathur [24]	*	*	*	*		*	*		**6**
Mansour [10]	*	*	*	*	*	*	*		**7**

Supplementary Table 3; Adverse events of corticosteroid therapy in tuberculous pleurisy

Study	Year	Side effects (cases)
Aspin [23]	1958	Rebound phenomenon (n=1)
Menon [8]	1964	Transient moon face (a few cases)
Mathur [24]	1965	No side effect
Lee [20]	1988	Epigastralgia, edema, transient moon face(n=1)
Galarza [9]	1995	No side effect
Wyser [22]	1996	Epigastric pain (n=4)
Bang [18]	1997	Side effect (n=1)
Lee [21]	1999	No side effect
Elliott [19]	2004	Hyperglycemia (n=2) Hypertension (n=3) Ative herpeszoster (n=3) Esophageal candidiasis (n=1)
Mansour [10]	2006	Not state
