# Supplementary material for: The efficacy and safety of adjunctive corticosteroids in the treatment of tuberculous pleurisy: a systematic review and meta-analysis
Source: Oncotarget. 2017 May 24;8(47):83315–22. doi: 10.18632/oncotarget.18160 (PMC5669971; doi:10.18632/oncotarget.18160)
Supplement: Supplementary file 2 [file oncotarget-08-83315-s002.docx]

**Additional File 1: Search strategies: A list of included and excluded articles and reasons of exclusion.**

**Trials were excluded if they did not meet with the following inclusion criteria. Figure 1 shows the reasons for the exclusion of various studies.**

The following inclusion criteria were used for determining which trials to use in the study: 1) trials that compared corticosteroids with a control (placebo or no steroids); 2) trials that enrolled tuberculous pleurisy cases; and 3) trials that reported results on residual fluid, pleural thickening, pleural adhesions, clinical symptoms, adverse events, and death. Two independent investigators (AP and SX) assessed each inclusion trial and extracted the data, such as trial characteristics and outcome measures (e.g., pleural thickening, pleural adhesions, adverse events, *p* values, response rate [RR] for residual fluid, 95% confidence interval [CI], and weighted mean difference [WMD] for number of days of clinical symptoms). The quality of RCTs was assessed using the Jadad scale, [11] and the non-RCT studies were estimated using the 9-star Newcastle–Ottawa Scale. [12]

**A list of excluded articles and reasons of exclusion:Language limited (n=4):**

1. Singh D, Yesikar SS. Role of intrapleural corticosteroids in tuberculous pleural effusion: a clinicotherapeutic trial of [50 cases.](http://paper.pubmed.cn/66a1ob2712) J Indian Med Assoc. 1965; 45:306–9.

2. Starostenko EV, Novoselova VP. [Indications for the use of prednisolone in tuberculosis]. [Article in Russian]. Probl Tuberk. 1989; :44–7.

3. Tani P, Poppius H, Maekipaja J. Cortisone therapy for exudative tuberculous pleurisy in the light of a follow-up study. Acta Tuberc Pneumol Scand. 1964; 44:303–09.

4. Tanzj PL, Andreini E. [On therapeutic use of corticosteroids in pleuro-pulmonary tuberculosis]. [Article in Italian]. Arch Tisiol Mal Appar Respir. 1965; 20:331–57.

**2. By manual searches, only abstract.**

1. Filler J, Porter M. Physiologic studies of the sequelae of tuberculous pleural effusion in children treated with antimicrobial drugs and prednisone. Am Rev Respir Dis. 1963; 88:181–88.

2.  Paley SS, Mihaly JP, Mais EL, Gittens SA, Lupini B. Prednisone in the treatment of tuberculous pleural effusions. Am Rev Tuberc. 1959; 79:307–14.

3. Commerford PJ, Strang JI. Tuberculous pericarditis. In: Coovadia HM, Benatar SR, (eds). A century of tuberculosis: South African perspectives. Cape Town: Oxford University Press; 1991. pp. 123–36.

**3. References to studies excluded from this review: Trials were excluded if they did not meet with the inclusion criteria. We list** 16 **references for examples:**

1. Bilaçeroğlu S, Perim K, Büyükşirin M, Celikten E. Prednisolone: a beneficial and safe adjunct to antituberculosis treatment? A randomized controlled trial. Int J Tuberc Lung Dis. 1999; 3:47–54.
2. Cherednikova GV. [Immediate and late results of treatment with corticosteroid hormones of children with tuberculosis]. [Article in Russian]. Probl Tuberk. 1973; 51:46–49.
3. Cisneros JR, Murray KM. Corticosteroids in tuberculosis. Ann Pharmacother. 1996; 30:1298–303.
4. Damany SJ, Shah KT. Treatment of pleural effusion with and without triamcinolone in addition to usual antituberculosis chemotherapy. J Indian Med Assoc. 1968; 51:391–93.
5. Fleishman SJ, Coetzee AM, Mindel S, Berjak J, Lichter AI. Antituberculous therapy combined with adrenal steroids in the treatment of pleural effusions: a controlled therapeutic trial. Lancet. 1960; 1:199–201.
6. Grewal KS, Dixit RP, Sil DR. A comparative study of therapeutic regimens with and without corticosteroids in the treatment of tuberculous pleural effusion. J Indian Med Assoc. 1969; 52:514–16.
7. Khomenko IS, Chukanov VI, Gergert VI, Utkin VV. [Effectiveness of antitubercular chemotherapy combined with corticosteroids and immunomodulators]. [Article in Russian]. Probl Tuberk. 1990; 1:24–28.
8. Manresa F, Galarza I, Cañete C. Using corticosteroids to treat tuberculous pleurisy. Chest. 1997; 112:291–92.
9. Mathur KS, Prasad R, Mathur JS. Intrapleural hydrocortisone in tuberculous pleural effusion. Tubercle. 1960; 41:358–62.
10. Mayanja-Kizza H, Jones-Lopez E, Okwera A, Wallis RS, Ellner JJ, Mugerwa RD, Whalen CC, and Uganda-Case Western Research Collaboration. Immunoadjuvant prednisolone therapy for HIV-associated tuberculosis: a phase 2 clinical trial in Uganda. J Infect Dis. 2005; 191:856–65.
11. Pacheco CR, Valdez-Ochoa S, Naranjo F, Alvarez H, Aguilar M, Saavedra M. [Clinical study of a new synthetic steroid in the treatment of pleural tuberculosis]. [Article in Spanish]. Gac Med Mex. 1973; 106:249–55.
12. Porsio A, Borgia M. [Controlled clinical trials of the use of a new anabolic agent in a Sanatorium]. [Article in Italian]. Clin Ter. 1966; 37:502–18.
13. Tuberculosis Research Centre. Study of chemotherapy regimens of 5 and 7 months’ duration and the role of corticosteroids in the treatment of sputum-positive patients with pulmonary tuberculosis in South India. Tubercle. 1983; 64:73–91.
14. Batungwanayo J, Taelman H, Allen S, Bogaerts J, Kagame A, Van de Perre P. Pleural effusion, tuberculosis and HIV-1 infection in Kigali, Rwanda. AIDS. 1993; 7:73–79.
15. Blumberg HM, Burman WJ, Chaisson RE, Daley CL, Etkind SC, Friedman LN, Fujiwara P, Grzemska M, Hopewell PC, Iseman MD, Jasmer RM, Koppaka V, Menzies RI, et al, and American Thoracic Society, Centers for Disease Control and Prevention and the Infectious Diseases Society. American Thoracic Society/Centers for Disease Control and Prevention/Infectious Diseases Society of America: treatment of tuberculosis. Am J Respir Crit Care Med. 2003; 167:603–62.
16. Engel ME, Matchaba PT, Volmink J.Corticosteroids for tuberculous pleurisy. Cochrane Database Syst Rev. 2007; :CD001876.
